# Analysis of potential risks of clinical application of Yi Dian Hong and its proprietary Chinese medicines: A review

**DOI:** 10.1097/MD.0000000000036860

**Published:** 2024-01-26

**Authors:** Gongzhen Chen, Leiming Mao, Huyan Xia, Lei Zhu, Jiamin Huang, Yingmin Lu, Xin Liu, Ting Tang

**Affiliations:** a First Clinical Medical College, Guizhou University of Traditional Chinese Medicine, Guizhou, China; b Guizhou University of Traditional Chinese Medicine, Guizhou, China; c Guiyang Second People’s Hospital, Guizhou, China.

**Keywords:** pyrrolizidine alkaloids, toxicity, Yi Dian Hong, Yi Dian Hong proprietary Chinese medicine

## Abstract

Yi Dian Hong, belonging to the Asteraceae family, finds widespread use in traditional Chinese medicine for its effectiveness in clearing heat, detoxifying, promoting blood circulation, reducing swelling, and cooling the blood. Modern medical research has revealed that Yi Dian Hong and its proprietary Chinese medicines possess biological functions such as inhibiting tumor-specific angiogenesis and regulating immune-related molecules. However, studies have identified that the primary component of Yi Dian Hong contains pyrrolizidine alkaloids (PAs), a toxic substance with potential risks to the liver, lungs, genes, and a propensity for carcinogenicity. Many countries impose strict controls on the content of PAs in herbal medicines and products. Unfortunately, China currently lacks relevant content standards, thereby introducing greater clinical application risks. To ensure the safety of clinical use of Yi Dian Hong, this review will analyze the risk associated with Yi Dian Hong and its proprietary Chinese medicines in clinical applications based on the PAs content in these medicines and provide recommendations.

## 1. Introduction

A small red plant, also known by various names such as goat foot grass, red under the leaves, red back leaves, red leaf grass, red back fruit, and stone green red, is found distributed across several provinces in China, particularly in South China, Southwest, and Southeast China, with its primary concentration in regions such as Sichuan, Yunnan, Hubei, Anhui, Guizhou, Hunan, and Zhejiang. Botanically, it belongs to the Asteraceae family, within the genus Emilia sonchifolia (L.) DC.^[1]^ Within this genus, Emilia sonchifolia (L.) DC., there are 8 Chinese patent medicines listed in the 2020 edition of the Chinese Pharmacopoeia that contain Emilia sonchifolia as a primary constituent. These medicines include Hua Hong Tablets (granules, capsule), Cold and Flu Inflammation and Cough Capsule (tablets), Lingyuan Wanying Tea, Bai Rong Cough Syrup, Bonesetters’ Oil, Injuries’ Oil, Wake-up-the-Spleen-and-Nourish-Children Capsule (granules), and Shaolin Bonesetter Cream.^[2]^ According to the Dictionary of Traditional Chinese Medicine,^[3]^ a small red plant is classified as a herbal remedy with heat-clearing, toxin-removing, anti-inflammatory, and diuretic effects. In clinical practice, it is commonly utilized for the treatment of conditions such as enteritis, dysentery, urinary tract infections, upper respiratory tract infections, conjunctivitis, oral ulcers, and canker sores. Its chemical composition primarily comprises alkaloids, flavonoids, organic acids, and terpenes, with PAs being one of its major alkaloid components.^[4]^PAs are a prevalent endogenous hepatotoxic component in the Asteraceae family, known for their significant hepatotoxicity, pulmonary toxicity, genotoxicity, and carcinogenicity. The hepatotoxicity of PAs has raised substantial concerns.^[5]^ Globally, relevant authorities and health organizations, including the World Health Organization (WHO), the European Union (EU), the United States (US), Germany, the United Kingdom (UK), and others, have implemented restrictions on the use of PAs. However, in China, only limitations on Senkirkine have been specified, while other traditional Chinese medicines and proprietary Chinese medicines containing PAs remain unrestricted. This poses a considerable challenge to clinical safety and human health. Similarly, there are no clear recommendations or risk warnings associated with the use of Yi Dian Hong and its proprietary Chinese medicines, which are frequently employed for various ailments in clinical practice. Consequently, this paper aims to analyze the toxicity and mechanisms of PAs, along with the content of PAs in Yi Dian Hong and its proprietary Chinese medicines. The objective is to elucidate the risks associated with their clinical use, providing a scientific foundation for assessing the safety of these drugs in clinical practice.

## 2. PAs conformational relationships

PAs are widely distributed plant toxins, and their constituents can enter the human body through various sources, including traditional herbs, teas, cereals, functional foods, and the food chain. This has raised significant concerns among global food and drug regulatory and research organizations.^[6,7]^The ingestion of PAs can result in poisoning, and in severe cases, it can lead to fatalities. It has garnered substantial attention from regulatory and research bodies worldwide. Acute poisoning caused by PAs can result in severe hepatotoxicity and may be accompanied by hemorrhagic necrosis, leading to conditions such as hepatic sinusoidal obstruction syndrome (HSOS).^[8]^ Chronic poisoning primarily affects the liver, lungs, and blood vessels, and occasionally impacts organs such as the kidneys, pancreas, gastrointestinal tract, bone marrow, and brain. Research has demonstrated that prolonged exposure to chronic toxicity can cause significant harm, including hepatic and pulmonary venous occlusion, steatosis, loss of metabolic function, biliary epithelial hyperplasia, cirrhosis, adenomas, or even cancer.

**Figure 1.**
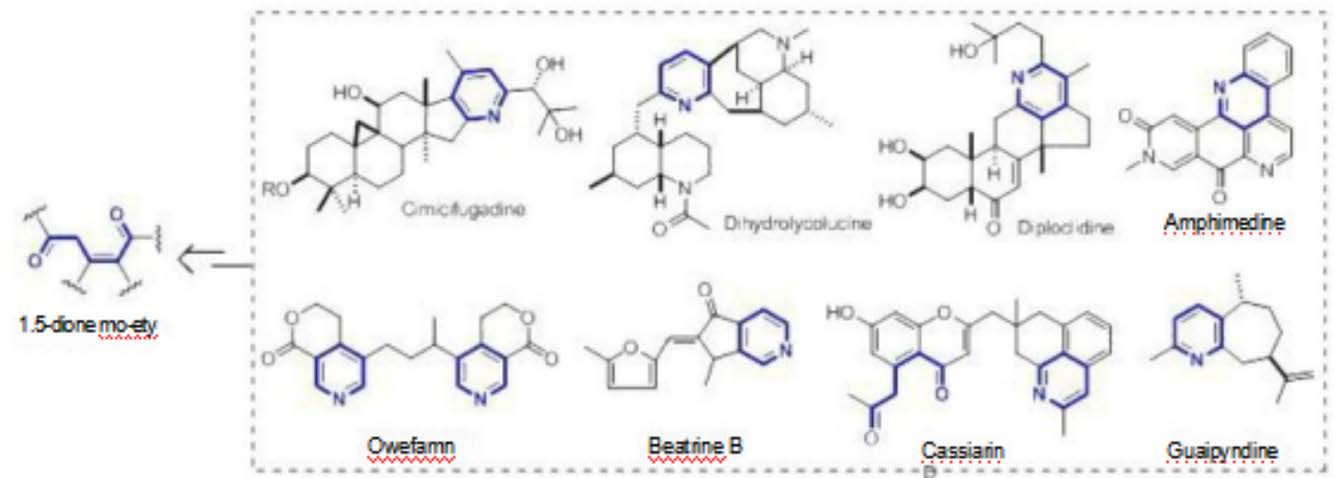
Biosynthesis of pyridine alkaloids.

The molecular structure of PAs primarily consists of necine base and necic acid, forming esters. PAs can be categorized into 2 main groups: saturated PAs and unsaturated PAs. Unsaturated PAs are considered toxic due to the presence of 1,2-unsaturated double bonds. PAs can be further classified into 21 different species based on the structure of the chiridonium substituent. Out of these, 5 PAs contain unsaturated bonds, belonging to structural types such as retronecine (inverted chiricobaltic alkaloid type), otonecine (osso chiricobaltic alkaloid type), heliotridine (aspergillus alkaloid type), crotanecine, and supinidine (supine aspergillus alkaloid type). Among these, retronecine and otonecine are the most commonly encountered types. Additionally, retronecine is often stored in plants in the form of N-oxide.^[6]^ Ester structures represent a common category of compound structures, encompassing various types, including ester-free substitutions, monoester substitutions at the 7- or 9-position, 7,9-diester substitution ring-openers, 11-membered macrocyclic bis-esters, 12-membered macrocyclic bis-esters, 13-membered macrocyclic bis-esters, and others. It has been observed that the key structural features contributing to the toxicity of esters are the presence of a 1,2-position double bond within their molecules and the esterification of at least one of the hydroxyl groups at the 7- or 9-position. It noteworthy that ester compounds meeting these 2 conditions tend to exhibit strong hepatotoxicity, earning them the designation of hepatotoxic pyrrolizidine alkaloids (hepatotoxic PAs, HPAs). Examples of HPAs include retronecine-type monocrotaline and otonecine-type clivorine (Fig. 1).

As per China Pharmacopoeia, “Yi Dian Hong” contains PAs species including Kirschner chirimenidine, lithosamine, inverted chirimenidine, and Euthanasia mustard alkaloids.^[8,9]^ Research has demonstrated that PAs containing chirimenidine have profound hepatotoxic effects, resulting in liver injury. Consequently, the issue of hepatotoxicity induced by PAs-associated herbal medicines has garnered significant attention on the global stage. It has emerged as one of the most critical challenges in the realm of drug-induced liver injury within traditional Chinese medicine today.

## 3. Mechanism of pyrrolizidine alkaloid toxicity

Pyrrolizidine alkaloids (PAs) exhibit severe toxic effects in both humans and animals, encompassing hepatotoxicity, pulmonary toxicity, genotoxicity, neurotoxicity, and embryotoxicity.^[9]^ The extent of these toxicities is intricately linked to the structural characteristics and properties of PAs and is influenced by the species or individual differences within the animal kingdom. Therefore, the manifestation and severity of these toxicities can vary significantly depending on the specific PAs involved and the species or individual under consideration.

## 4. Mechanisms of hepatotoxicity

The liver is the primary target for PAs-induced toxicity due to the fact that bioactivation primarily takes place in the liver. HSOS stands out as the most common clinical manifestation and is considered the hallmark of PAs poisoning. PAs poisoning can be categorized into 3 main types: acute, subacute, or chronic, each presenting with distinct symptoms.Acute poisoning is characterized by features such as hemorrhagic necrosis, hepatomegaly (enlarged liver), and ascites (abdominal fluid accumulation). The hepatic venous obstruction, leading to HSOS, is a prominent feature of acute PAs poisoning.^[10,11]^ Chronic PAs exposure is marked by biliary epithelial necrosis, fibrosis, cirrhosis (advanced liver scarring), and hyperplasia (abnormal cell growth). Liver failure and death are the ultimate outcomes associated with chronic PAs poisoning.^[12,13]^ Liver failure and death represent the most severe levels of PAs-induced toxicity, underscoring the critical importance of understanding and managing PAs-related health risks.

**Figure 2.**
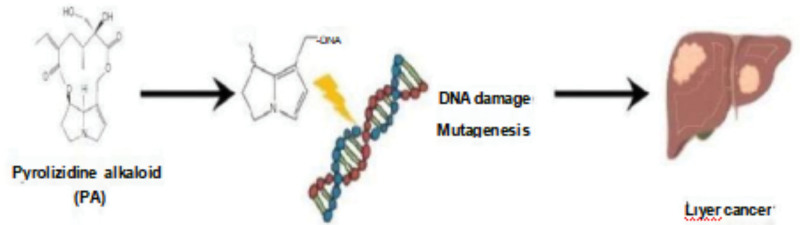
Hepatotoxicity of pyrrolizidine alkaloids.

Research has definitively established that the toxicity of PAs is intricately linked to their specific pyrrole metabolism.^[14]^ Once these PAs molecules enter an organism, they undergo metabolic dehydrogenation, transforming into dehydropyrrolizidine alkaloids (DHPA) under the influence of cytochrome P450 (CYP) 3A enzymes. DHPAs are highly electrophilic and readily form adducts with intracellular nucleophilic substances such as DNA, RNA, and proteins. This interaction leads to cellular damage and the initiation of apoptosis in hepatocytes.CYP3A is primarily found within the intracellular plasma, particularly on the rough endoplasmic reticulum surface. Consequently, for PAs to exert their toxic effects, they must first penetrate the cell. Organic cation transporters play a crucial role in facilitating the hepatic uptake of PAs, working in conjunction with CYP3A to contribute to PAs-induced hepatotoxicity.^[15]^ Unsaturated dehydropyrrole metabolites produced during PAs metabolism within the body can form complexes with macromolecules, including proteins. This interaction results in hepatocyte necrosis and subsequent hepatotoxicity.^[16]^ Furthermore, oxidative damage plays a significant role in the toxic effects of PAs.^[17]^ Oxidative stress induced by PAs metabolism further contributes to the overall toxicity associated with these compounds. Presently, 3 primary mechanisms underlie the hepatotoxicity of PAs:PAs trigger an overproduction of reactive oxygen species, inducing cellular oxidative stress. Specifically, isoline (retronecine-type) and clivorine (otonecine-type) have been implicated in affecting the activity of antioxidant enzymes within liver cells upon entering the liver. This disruption of antioxidant enzyme activity leads to cellular oxidative stress.^[18,19,20]^ Another mechanism involves the promotion of apoptosis via the mitochondrial intrinsic pathway. PAs can form pyrrole-ATP 5 B adducts in different types of hepatocytes, reducing intracellular ATP levels and disrupting mitochondrial homeostasis. This disruption, in turn, triggers mitochondrial dysfunction and cellular apoptosis.^[19]^ The third pathway of PAs toxicity is related to hepatic bile acid stagnation. 1,2-unsaturated PAs are known to decrease the gene expression of various hepatobiliary transport proteins, enzymes involved in bile acid synthesis and binding, and several transcriptional regulators. This disruption leads to the imbalance of bile acid homeostasis, ultimately causing liver injury.^[21]^ These 3 mechanisms collectively contribute to the hepatotoxicity associated with PAs exposure. Understanding these pathways is crucial for assessing and managing the risks posed by PAs-containing substances (Fig. 2).

## 5. Mechanisms of genotoxicity and tumourigenicity

In the mid-20th century, Schoental et al conducted experimental animal studies and discovered that retrosine could accumulate in various organs, including the liver, lungs, bladder, skin, and gastrointestinal tract. Importantly, retrosine had the capacity to induce tumors.^[22]^ Further research by Schoental et al confirmed the tumorigenic effects of retrosine and similar PAs in animal studies, particularly those belonging to the heliotridine, retronecine, and otonecine types. These studies highlighted the carcinogenic potential of certain PAs. Yang et al elucidated the mechanism by which riddelline (a retronecine-type PA) induces genotoxicity. This mechanism involves the formation of DHPA-derived DNA adducts in the liver, which contribute to tumorigenesis.^[23,24]^ Additionally, these compounds have the ability to react with proteins, leading to DNA cross-linking, sister chromatid exchanges, and chromosomal aberrations.^[25–27]^ The presence of DHPA-DNA adducts has been detected in tumor patients, and there is a strong correlation between the level of DHPA-DNA adducts and tumorigenicity.^[28,29]^ Furthermore, the level of DNA adducts induced by riddelline is associated with the development of hepatic angiosarcoma.^[30]^Moreover, PAs can induce hepatic tumors in rodents through the formation of DNA adducts. Importantly, the results of in vivo and in vitro mechanistic studies in experimental rodents have high relevance to humans,^[31]^ suggesting that riddelline can be genotoxic and tumorigenic in humans due to the formation of DHPA-derived DNA adducts.^[32]^ The metabolic activation of these tumorigenic PAs to form the same DHPA-derived DNA adducts implies that these adducts have the potential to serve as biomarkers for PAs-induced tumorigenicity.^[33]^In addition to their role in tumorigenesis, PAs have also been associated with skin cancer. This association arises from the consumption and metabolism of PAs in animals, which can lead to photosensitization.^[34,35]^

## 6. Mechanisms of pulmonary toxicity

The lungs can also become a target for injury from PAs, as DHPA has the capacity to enter small pulmonary arteries from the liver, resulting in injury akin to hepatic sinusoidal obstruction syndrome. Once present in the lungs, DHPA can lead to thrombus formation and thickening of the vessel walls, ultimately causing vessel occlusion and inflammation.^[36,37]^ The combination of these effects triggers pulmonary arterial hypertension (PAH), which can subsequently lead to congestive heart failure. Culvenor et al’s study suggests that this phenomenon is associated with low levels of PAs (0.025 mmol/kg body weight) and prolonged exposure.^[38]^ This extended exposure resulted in 2 distinct types of lung lesions: one characterized by intravascular aggregates of single nucleated cells, ultimately leading to venous occlusion; and the other exhibiting extravascular changes, including thickening of the alveolar septa and an increased cell count. In a clinical case report, a 66-year-old woman developed progressive dyspnea after long-term consumption of herbal tea containing comfrey ingredients and was diagnosed with PAH.^[39]^ This case underscores the potential health risks associated with prolonged exposure to PAs, especially in herbal products.

## 7. Other toxicity

Certain PAs have the ability to breach the placental barrier and enter the fetal circulation, raising concerns about their potential embryotoxicity.^[40]^ There have been instances where a pregnant woman consumption of herbal tea containing PAs led to neonatal HSOS.^[41]^ Animal studies have further illustrated the adverse effects of PAs on pregnancy, including fetal weight loss, teratogenicity, preterm delivery, and even fetal mortality in rats exposed to PAs during early pregnancy.^[41]^

Collectively, the research suggests that PAs can inflict various forms of irreversible toxic damage on the organism, with hepatotoxicity being a prominent concern. Despite significant advancements in medical technology in recent decades, there remains a lack of effective clinical treatments for severe conditions such as PAs-induced HSOS.^[42]^ Consequently, there is an urgent need for extensive and in-depth research on PAs toxicity and the development of effective treatment strategies.

## 8. Analysis of the risks and factors of clinical application of Yi Dian Hong and its proprietary Chinese medicines

### 8.1. Risks of clinical application of Yi Dian Hong and its pCms

Numerous plants belonging to different families and regions contain PAs. In addition to well-studied ones like Qianlong, Fenghuang, Pelargonium, Rehmannia, and Noma, there are lesser-known Chinese herbal medicines, such as Yi Dian Hong and Dian Zicao, that also contain PAs. Yi Dian Hong, in particular, has a broad range of clinical applications. It is not only used in traditional prescriptions but also serves as a primary ingredient in various proprietary Chinese medicines listed in the Pharmacopoeia. Some of these include Huahong tablets, Huahong granules, Huahong capsules, cold and inflammatory cough capsules, Lingyuan Wanying tea, white velvet cough syrup, bone-setting oil, injury oil, cold and inflammatory cough tablets, Huahong capsules, and products for waking up the spleen and nourishing children, among others.The applications of Yi Dian Hong span multiple medical disciplines and systems, including internal medicine, pediatrics, orthopedics, oncology, and more. Furthermore, it comes in various dosage forms such as tablets, granules, capsules, syrups, etc, enabling both internal and external treatment. It evident that Yi Dian Hong has a wide range of applications. However, it is concerning that the instructions for the above-mentioned proprietary Chinese medicines do not contain information about the risks, precautions, and contraindications associated with PAs. This omission poses a significant potential danger in the clinical use of these medications. Addressing these risks and providing clear guidance is crucial for patient safety. Several cases of toxicity related to the clinical application of Yi Dian Hong-related drugs have been documented.

In a case study by Deng Zeping, a 44-year-old female patient who had been self-administering Yi Dian Hong root and leaf (approximately 100g/day) for 3 weeks was admitted to the hospital with symptoms of “abdominal distension for 10 days.” Subsequently, she developed hepatomegaly, ascites, liver congestion, and liver function impairment. The cause was attributed to PAs present in Yi Dian Hong, leading to HSOS. Another case by Chen Ruanqin reported a 54-year-old male patient who was admitted to the hospital with symptoms of “fatigue, abdominal distension for 2 months, yellow eyes, yellow urine, and scanty urine for 3 weeks.” This patient had been consuming herbal tea containing Yi Dian Hong for 9 months and subsequently developed jaundice, ascites, and hepatic dysfunction. The PAs in Yi Dian Hong were identified as the cause of HSOS in this patient. According to the Pharmacopoeia, Doronine and Senkirkine are the primary PAs found in Yi Dian Hong. The cases mentioned above underscore the widespread use of Yi Dian Hong and its proprietary Chinese medicines in China. They also highlight the severe adverse reactions that can result from the PAs in these substances, with poor prognoses and high risks associated with their use. Therefore, it is of paramount importance to formulate and use Yi Dian Hong and related proprietary Chinese medicines in a reasonable and correct manner to achieve optimal therapeutic effects without causing harm to the patient.

## 9. Risk factor analysis of Yi Dian Hong and its pCms

In recent years, the toxicity of PAs has garnered significant attention from both domestic and international authorities. Consequently, various health and drug regulatory agencies, including the World Health Organization (WHO), the European Union, the United States, Germany, and others, have implemented strict restrictions on the usage and dosage of PAs.

The WHO has recommended the monitoring of pyrrolizidine alkaloid levels in honey and dairy products and has issued guidelines pertaining to the health and safety considerations related to PAs. The European Commission has established regulations stipulating that PAs-like components should not be detectable in blue thistle oil (primarily derived from the seed oil of the plantain-leaf blue thistle Echium plantagineum L., from the Comfreyaceae family) used as an ingredient or additive in food products, with a limit of detection set at 4 ppb. Furthermore, the EU has adopted a “zero tolerance” policy for substances lacking a medicinal risk assessment or whose safety cannot be substantiated based on available research information. This principle has been applied for the first time in controlling PAs in blue thistle oil. The US. Food and Drug Administration has prohibited the food processing industry from utilizing plants belonging to the Polygonum (Symphytum) genus of the comfrey family as ingredients. It is evident that foods and medicines containing PAs have permeated various aspects of people lives. The potential toxic hazards they pose should not be underestimated, as they represent a substantial threat to human health and safety.

In 1992, German health authorities established an Acceptable Daily Intake for herbal products containing pyrrolizidine alkaloids (PAs). According to these regulations:

- The intake of PAs and their N-oxides should not exceed 1 μg.
- For long-term use (more than 6 months), the limit is set at 0.1 μg.
- For topical herbal products, the limit is 10 μg.
- PAs-containing products are prohibited for use by pregnant and lactating women.

Similarly, food authorities in New Zealand and Australia have specified that the daily intake of PAs per person should not exceed 1 μg/kg.

Additionally, relevant authorities in the Netherlands have established a limit where the total PAs content should not exceed 1 ppb (equivalent to 1 μg) per 1 kg or 1 L of herbal products or extracts. The European Medicines Agency conducted comprehensive testing in 2016 and identified 28 hepatotoxic pyrrolizidine alkaloids (HPAs) using solid phase extraction-high performance liquid chromatography-tandem mass spectrometry (SPE-HPLC-MS/MS). They set the most stringent regulation on HPAs, specifying that the daily intake should not exceed 0.007 μg/kg. Due to the high toxicity hazard associated with PAs, countries around the world have implemented specific standards and limits to mitigate or prevent the harm they may cause to human health.

In our nation, current regulations and standards pertaining to PAs are notably scarce. Chirimenoside, a commonly utilized medicinal herb, has been subject to limited scrutiny within the Chinese Pharmacopoeia, primarily for its constituent HPAs. As per the Pharmacopoeia, only the marker compound, adonifolinine, has been identified, with a stipulated content not exceeding 0.004% of the dried product. According to the Pharmacopoeia, the recommended dosage for Trigonelline is 1530 grams, allowing for a daily intake of up to 600 to 1200 micrograms, based on the established threshold value for qualified herbs. When calculated based on an adult body weight of 60kg, this equates to a daily intake of 10 to 20 micrograms per kilogram of body weight. These regulations align with the 1989 WHO guidelines. It is essential to highlight that, aside from Trigonella foetidis herbs, there exists no pertinent quality control or risk indication for other herbs such as Yi Dian Hong, Comfrey, Fenugreek, Chrysanthemum Panax, and proprietary Chinese medicines containing Trigonella foetidis as their primary constituent, such as Qianbai Rhinitis Tablets and Qianxi Tablets. Consequently, particular attention must be directed toward assessing the quality and potential risk factors associated with the utilization of these herbs and medicines.

In the realm of multidisciplinary clinical medicine, only a subset of the aforementioned Yi Dian Hong pCms includes information on the proportion of PAs they contain. In the 2020 edition of the Pharmacopoeia, Huahong Tablets, Huahong Granules, and Huahong Capsules are specified to contain 18% PAs. The Cold Inflammatory Cough Syrup from the Ministry of Traditional Chinese Medicines (PCM) contains 35.56% PAs, Lingyuan Wanying Tea contains 1.35%, White Velvet Cough Syrup contains 18.8%, and White Velvet Cough Syrup contains 17.84%. Furthermore, the Compendium of pCms lists Hua Hong Capsule with a PAs content of 17.84%.While these products indicate the PAs content, their instructions fail to specify the precise amount of Yi Dian Hong herb used, making it challenging to determine compliance with the WHO regulations, which stipulate that PAs should not exceed a daily intake of 0.015 mg/kg^−1^. The drug instructions also lack information regarding the risks associated with their use and precautionary measures. Some medications offer only a simple cautionary note advising patients with liver and kidney diseases to use the drug under the guidance of a physician. It is crucial to note that most Yi Dian Hong pCms are available over-the-counter, enabling patients to purchase and self-administer them. Additionally, a significant concern arises from the fact that many of these products are consumed by children, presenting a substantial challenge to the safety of pediatric medication. Beyond clinical use, some patients directly prepare these herbs for tea consumption, potentially leading to inadvertent overconsumption of PAs, which can result in acute or chronic poisoning.

## 10. Suggestions for clinical use of Yi Dian Hong and its proprietary Chinese medicines

The extensive utilization of Yi Dian Hong and pCms containing Yi Dian Hong has introduced significant challenges to the safety of medication in clinical practice. To ensure a safer and more rational use of Yi Dian Hong and its associated pCms, the following recommendations are proposed, aiming to establish a solid foundation for the secure administration of these medicines in clinical settings.

## 11. Restriction of PAs intake and dosing range

To enhance the safety of Yi Dian Hong and pCms containing it, it is essential to establish limitations on daily and cumulative exposure to pyrrolizidine alkaloids (PAs) as follows: In 1898, a minimum daily intake of PAs in humans capable of causing hepatic sinusoidal obstruction syndrome (HSOS) was established at 15 µg/kg^−1^.Germany introduced PAs limits in 1992. The daily intake of unsaturated PAs is restricted to 1 µg for internal use and 100 µg for external use. The duration of use for PA-containing drugs should not exceed 6 weeks per year.The United Kingdom established a Tolerable Daily Intake (TDI) of 0.1 µg/kg^−1^ for PAs in 2003.The Netherlands also adopted a TDI of 0.1 µg/kg^−1^ for PAs in 2005.To address the carcinogenicity of PAs, the UK proposed a TDI of no more than 0.007 µg/kg for unsaturated PAs in 2008. The EU and Germany also endorsed this limit in 2011 and 2013, respectively. The EU proposed a solution for PAs contamination in 2016, with a 3-year transition period. During this period, the limit for PAs is 1 µg/day^−1^. If the contamination issue remains unresolved, the limit will be reduced to 0.35 µg/day^−1^.In line with the IPCS Health and Safety Guideline No. 26: Health and Safety Guidelines for Pyrrolizidine Alkaloids issued by the World Health Organization (WHO) in 1989, it is recommended that daily PA intake should not exceed 0.015 mg/kg^−1^. Based on this recommendation, for Yi Dian Hong herbs, it is advised that the intake of dried product should not exceed 20 g per day, and for fresh product, it should not exceed 30 g. A study involving 13 batches of Yi Dian Hong herb samples found that the average PAs intake in these samples ranged from 0.15 to 0.81 mg/g.

Secondly, it is imperative to restrict the scope of usage. Various countries have implemented measures to limit the use of herbal medicines containing pyrrolizidine alkaloids (PAs):The German Federal Office of Health mandates that all herbal medicines containing PAs must adhere to specified PAs content levels and be labeled as “not suitable for pregnant and breastfeeding women.” Consequently, the sale and utilization of numerous herbal products containing PAs have been halted. Switzerland and Austria have similarly enacted regulations for herbal medicines, aligning with the German approach. The US Food and Drug Administration prohibited the use of comfrey containing harmful PAs in dietary supplements in 2001. In January 2004, the United Kingdom called for a halt to the sale of oral products containing harmful PAs. The American Herbal Products Association and the Consumer Healthcare Products Association have recommended the addition of warning labels to herbal medicines containing harmful PAs. These labels may include instructions such as “For external use only,” “Do not use on broken skin,” and “Contraindicated during breastfeeding.”

Moreover, proprietary Chinese medicines like Qianbai Rhinitis Tablets are also proscribed for use by pregnant and lactating women, children, and the elderly. In clinical practice, special attention should be paid to the potential risk of hepatotoxicity associated with PAs-containing herbal medicines. Such products should be either avoided or used with caution in specific populations, with enhanced monitoring of liver function. Moreover, it is crucial to provide clear warnings regarding the associated risks of using Chinese medicines containing PAs. To ensure patient safety, instruction manuals for these medicines should include explicit information indicating the potential for hepatotoxicity and other related risks. Patients should be cautioned against concurrent use of other medications that may pose a liver risk while taking these medicines, and they should be advised to abstain from alcohol consumption during this period. Combining alcohol with medicines that may synergistically enhance the hepatotoxic effects of PAs should also be strictly avoided. These warnings are designed to safeguard liver health and prevent unnecessary risks. Therefore, when patients use Chinese medicines containing PAs ingredients, it is essential for them to diligently follow their healthcare provider advice and remain cognizant of the relevant contraindications and usage precautions.

## 12. Strict quality control of PAs

Furthermore, it is imperative to establish corresponding regulations and a monitoring system to oversee the collection, distribution, and utilization of Yi Dian Hong herbs: Researchers,^[43]^ have conducted analyses and comparisons of 6 batches of Hedyotis diffusa harvested at different times in Guangxi. Their findings indicated that the alkaloid content in Hedyotis diffusa harvested in August was the highest. This information underscores the importance of understanding the variations in alkaloid content based on harvesting periods. To control and mitigate the transmission and accumulation of PAs, particularly HPAs, within the pharmaceutical or food supply chain, it is essential to bolster Good Agricultural Practices during cultivation and adhere to Good Manufacturing Practices in the production of Yi Dian Hong preparations. A comprehensive approach involving chemistry, biology, and pharmacology is required for in-depth research on Yi Dian Hong and its proprietary Chinese medicines, which are governed by current Pharmacopoeia Laws. Research should encompass fundamental principles, pharmacodynamic substance characterization, analytical methods, toxicity, and detoxification, among other aspects. The objective is to establish scientifically grounded and reasonable limit standards and testing methods. These standards and methods may vary depending on the herb variety and product grade, whether it is an herb, proprietary Chinese medicine, or compound preparation. The results of these studies should be incorporated into routine testing, toxicological composition analysis, and adverse reaction monitoring protocols for Yi Dian Hong and its proprietary Chinese medicines. A comprehensive analysis of the benefits and risks associated with Yi Dian Hong should be conducted, considering the principles of compounding, concoction, reduction of toxicity, and evidence-based treatment in Chinese medicine. Through thorough evaluation, potential hazards and risks to human health can be assessed, allowing for the development of proactive and objective countermeasures.

## 13. Discussion and outlook

As an increasing number of toxicity events related to PAs have come to light, there has been a growing focus on drugs containing PAs. These drugs can be broadly classified into 4 categories based on their toxicological effects: acute toxicity, chronic toxicity, genotoxicity, and specific toxicity. PAs primarily exert their toxic effects on the liver, but they can also harm other organs such as the lungs, heart, kidneys, pancreas, and brain. Additionally, they have been associated with clear carcinogenic, mutagenic, and teratogenic effects. To safeguard public health, countries worldwide have established stringent limits and regulations to control the use and exposure of PAs. However, it worth noting that China Pharmacopoeia currently provides dosage guidelines for Qianlong, with limited provisions addressing the clinical use and exposure of Yi Dian Hong and its proprietary Chinese medicines. While these medicines are widely employed in clinical practice and have demonstrated therapeutic efficacy, their clinical application carries a substantial risk due to the presence of PAs. Furthermore, the toxicity associated with PAs is often severe and can result in poor patient outcomes. This paper has conducted an analysis of the risk factors associated with Yi Dian Hong, aiming to lay the groundwork for future dosage regulations and the safe clinical utilization of this medication.

## Author contributions

**Conceptualization:** Huyan Xia.

**Formal analysis:** Lei Zhu.

**Investigation:** Jiamin Huang.

**Software:** Leiming Mao.

**Supervision:** Yingmin Lu, Xin Liu, Ting Tang.

**Validation:** Leiming Mao.

**Writing – original draft:** Gongzhen Chen, Leiming Mao.

**Writing – review & editing:** Gongzhen Chen, Leiming Mao.
